# Investigating the impact of wet rendering (solventless method) on PUFA-rich oil from catfish (*Clarias magur*) viscera

**DOI:** 10.1515/biol-2022-0903

**Published:** 2024-07-17

**Authors:** Jaydeep Dave, Ali Muhammed Moula Ali, Nishant Kumar, Muralidharan Nagarajan, Marek Kieliszek, Sri Charan Bindu Bavisetty

**Affiliations:** School of Food-Industry, King Mongkut’s Institute of Technology Ladkrabang, Bangkok, 10520, Thailand; Kantaben Kashiram Institute of Agricultural Sciences and Research, Ganpat University, Mehsana, Gujarat, 384012, India; Department of Food Science and Technology, National Institute of Food Technology Entrepreneurship and Management, Kundli, Sonipat, Haryana, 131028, India; Department of Fish Processing Technology, Tamil Nadu Dr. J Jayalalithaa Fisheries University, Dr. M.G.R Fisheries College and Research Institute, Ponneri, 601204, Tamil Nadu, India; Department of Food Biotechnology and Microbiology, Institute of Food Sciences, Warsaw University of Life Sciences – SGGW, Nowoursynowska 159 C, 02-776, Warsaw, Poland

**Keywords:** catfish waste management, fish oil new resource, solvent-free extraction, polyunsaturated fatty acids, fatty acid analysis, volatile compounds

## Abstract

Catfish (*Clarias magur*) is a popular freshwater fish food worldwide. The processing of this fish generates a significant amount of waste, mainly in the form of viscera, which constitutes around 10–12% of the fish’s total weight. This study was focused on extracting polyunsaturated fatty acid (PUFA)-rich oil from catfish viscera, aiming to enhance the extraction process and make the production of oil and handling of fish byproducts more cost-effective. The wet reduction method, a solvent-free approach, was used for extraction, with yield optimization done via the Box–Behnken design. The resulting oil was evaluated for its oxidative quality and chemical characteristics. The optimal conditions for the wet rendering process were as follows: viscera to water ratio, 1:0.5 (w/v); temperature, 90℃; and time, 20 min, yielding 12.40 g/100 g of oil. The oil extracted under optimal wet rendering conditions had quality and oxidative stability comparable to solvent extraction and fewer secondary oxidation compounds. This oil had a higher PUFA content, specifically a 4:1 ratio of omega 6 to omega 3. Such oil, derived from catfish viscera, is suitable for the food industry due to its solvent-free extraction method.

## 1 Introduction

Catfish is a freshwater fish popular among consumers worldwide, with a global production rate reaching 5.8 million metric tons/year in 2021 [1]. A massive increase in demand exists for catfish driven by the consumer’s need for sustainable sources of proteins and healthy lipids, low cost, and organoleptic properties [2]. Asia is the largest producer of catfish, and China is the topmost producer. The most farmed catfish for consumption are the channel catfish (*Ictalurus punctatus*) and the African catfish (*Clarias gariepinus*). The other type of catfish, *Clarias magur,* commonly found in South and Southeast Asia, is considered a delicacy in countries like Thailand, Indonesia, Vietnam, India, and Bangladesh [3,4]. *C. magur* is a relatively new species in aquaculture and has yet to be utilized by the processing industry [5]. Therefore, limited research has been conducted in developing value-added products from this fish or its processing byproducts.

Generally, fish processing generates significant quantities of waste (40–60%, based on wet weight) in the form of head, skin, scales, bones, trimmings, and viscera [6]. Typically, this waste is used to develop low-valued animal feed or fertilizers or discarded, which can contribute to environmental issues [7]. Fish processing waste, especially viscera, contains a significant amount of bioactive compounds, i.e., squalene, monounsaturated fatty acids (MUFA), and polyunsaturated fatty acids (PUFA) [8,9]. It has been reported that different species of catfish may contain 10–40% oil based on the weight of visceral biomass, with the composition including saturated and unsaturated fatty acids, omega 6, and omega 3 fatty acids [10]. Owning to their habitat in freshwater, catfish have lower levels of omega-3 fatty acids, i.e., eicosapentaenoic acid (EPA) and docosahexaenoic acid (DHA), as compared with marine fish oil [11]. While catfish still contain significant quantities of oleic acid, linoleic acid, and alpha-linolenic acid (ALA), which have been associated with numerous health benefits, including improved heart and brain health, maintained healthy skin, reduced inflammation, and lower cholesterol and triglycerides levels [12,13].

Typically, recovery of fish oil is done using various chemical solvents, enzymatic hydrolysis, supercritical fluid extraction, wet rendering, and cold pressing [14]. However, some of these methods are linked to several detriments. For instance, chemical solvent extraction can leave traces of hazardous chemicals and is not eco-friendly. Cold pressing, while solvent-free, yields very low amounts of oil. Enzymatic and supercritical fluid extraction, despite being effective, are associated with higher capital expenditures and are hence not cost-effective. Dry rendering, another solvent-free method, involves cooking the fish biomass at high temperatures without water, which can lead to the degradation of heat-sensitive nutrients and the development of off-flavors due to prolonged exposure to high heat [15]. Due to these considerations, the present research dwells on solvent-free, eco-friendly, and cost-effective methods for recovering fish oil [16,17]. In this regard, wet rendering is gaining interest, as it involves cooking fish biomass in water or steam. This method not only prevents the degradation of heat-sensitive nutrients but also minimizes the development of off-flavors, as the lower temperatures used in wet rendering are more gentle on the biomass. The oil released is easily separated from the water and other fish solids, making this process particularly efficient and environmentally friendly [18]. Taati et al. [19] found that wet rendering produced higher fish oil yields than other extraction methods, such as acid hydrolysis and solvent extraction.

Wet rendering is a simple method for fish oil extraction, but it produces relatively impure oil, which may contain residual water, protein, and contaminants. During wet rendering, several factors can affect the yield and quality of fish oil, including the ratio of water to biomass, cooking time, and temperature [20]. Studies show that varying temperatures and times significantly impact the extraction yields and quality of fish oil using the wet rendering process. For instance, using an autoclave at 121°C with a 30-min holding time optimized the oil extraction from yellowfin tuna heads, providing a balance in fatty acid content and minimizing oxidative damage [21]. Another study indicated that increasing extraction temperatures from 80 to 100°C increased oil yields from tilapia and mackerel viscera, with longer cooking times (up to 60 min) further enhancing oil recovery [22]. These variations underscore the necessity of optimizing temperature and time to maximize fish oil extraction efficiency and quality. Therefore, optimizing the cooking conditions can enhance the extraction process’s efficiency, cost-effectiveness, quality, and safety. Hence, this research aimed to optimize the wet rendering extraction to recover high-quality fish oil from catfish (*C. magur*) viscera. The effect of extraction on oil yield, quality, and oxidative stability was studied.

## 2 Materials and methods

### 2.1 Chemicals

Thiobarbituric acid (TBA), trichloroacetic acid (TCA), ammonium thiocyanate, ferrous chloride, and sodium hydroxide (NaOH) were purchased from Merck (Darmstadt, Germany). Solvents, including chloroform, methanol, and hexane, were obtained from Lab-Scan (Bangkok, Thailand). Supelco^®^ 37 component FAME mix (purity 96 to 99%), 1,1,3,3-tetramethoxypropane (purity >98%), and ammonium thiocyanate (purity >98%) were procured from Sigma-Aldrich (St. Louis, MO, USA). Cumene hydroperoxide (C₆H₅C(CH₃)₂OOH) and 2-TBA (C_4_H_4_N_2_O_2_S) were obtained from Fluka Co. (Buchs, St. Gallen, Switzerland). The rest of the chemicals were obtained from Lab-Scan Ltd. (Bangkok, Thailand). All the chemicals used were of analytical grade.

### 2.2 Raw material

Freshly available catfish viscera (including liver and intestines) were procured from the Huatake fish market at Ladkrabang, Bangkok, Thailand. Approximately 2–3 kg of viscera was packed in polyethylene bags, placed into the polystyrene container containing ice, and transported to the faculty of the food industry, KMITL. Upon arrival, the viscera were minced using a meat grinder (Model: HR271331, Philips, Netherlands), and the minced visceral samples were used for further analysis.

### 2.3 Proximate composition

The proximate composition, moisture, proteins, fats, and ash were determined using the AOAC method to be 930.15, 923.03, 920.39, and 923.01, respectively [23,24].

### 2.4 Extraction of oil

**Figure 1:**
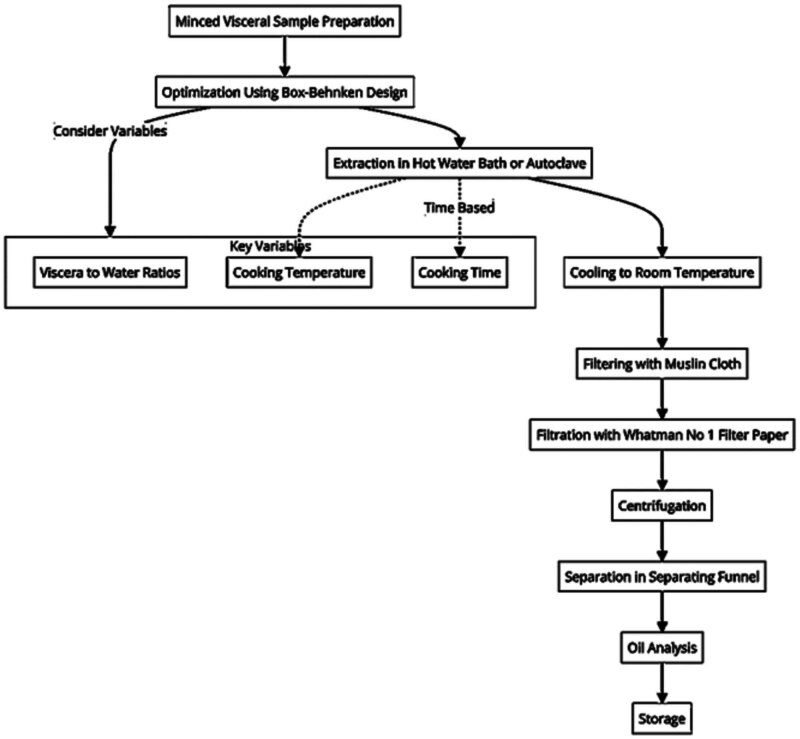
Process flow diagram for oil extraction from minced visceral samples using a BBD for optimization.

A minced visceral sample (100 g) was used for oil extraction by wet rendering method (solvent-free extraction). The extraction conditions were optimized using the Box-Behnken design (BBD); the experimental parameters are mentioned in Table 1. Three independent variables were considered, i.e., viscera to water ratios (1:0.5, 1:1.25, and 1:2, w/v), cooking temperature (50, 85, and 120℃), and cooking time (5, 15, and 25 min). The wet rendering was performed as suggested by Dave et al. [25] with slight modifications. The extraction was carried out in a hot water bath for 50 and 85℃, and in an autoclave for 120℃. After the experimental run, the sample temperature was brought down to room temperature. The extraction mixtures were filtered using a muslin cloth and filtrated using Whatman no 1 filter paper. The filtrate was further subjected to centrifugation at 2,500 × *g* for 15 min, the pellets (cell debris) were discarded, and the liquid samples were transferred to a separating funnel to separate oil from an aqueous phase. The recovered oil was subjected to analysis, the excess fat was transferred to an amber bottle, and headspace was flushed using nitrogen gas and stored at −20℃. Figure 1 depicts the flowchart for the extraction process.

**Table 1:** Runs showing various factors of response surface methodology for oil extraction using the wet rendering method obtained through BBD

Run	Factor A, viscera:water (w/v)	Factor B, temperature (℃)	Factor C, time (min)
1	1:1.25	85	15
2	1:1.25	85	15
3	1:2.00	85	5
4	1:2.00	50	15
5	1:0.50	85	25
6	1:1.25	120	5
7	1:2.00	85	25
8	1:1.25	85	15
9	1:0.50	85	5
10	1:1.25	120	25
11	1:1.25	50	5
12	1:0.50	50	15
13	1:0.50	120	15
14	1:1.25	50	25
15	1:2.00	120	15

The oils extracted using solvent methods, i.e., chloroform–methanol (2:1) mixture and hexane, were considered control [8].

### 2.5 Extraction yield

The obtained oil was used to determine the extraction yield. The extraction yield and oil recovery were calculated according to Dave et al. [25]. The yield in terms of g/100 g, wet basis, was calculated using the following formula:

(1) \[{\mathrm{Yield}}({\mathrm{g}}\left/100{\mathrm{g}})=\frac{{\mathrm{Weight\; of}}{\mathrm{the}}{\mathrm{obtained\; oil}}({\mathrm{g}})}{{\mathrm{Weight\; of}}{\mathrm{the}}{\mathrm{sample}}{\mathrm{used}}{\mathrm{for\; the\; extraction}}({\mathrm{g}})}\times 100.]\]

The oil recovery in terms of percentage was calculated using the following formula:

(2) \[{\mathrm{Oil\; recovery}}(\left \% )=\frac{{\mathrm{Weight\; of}}{\mathrm{the}}{\mathrm{obtained\; oil}}({\mathrm{g}})}{{\mathrm{Total\; oil\; present\; in\; the\; sample}}({\mathrm{g}})}\times 100.]\]

### 2.6 Oil quality

#### 2.6.1 Color

The color of the extracted oils was determined using a HunterLab colorimeter (LabScan® XE, Reston, VA, USA) and reported in the CIE color system (L*, a*, and b* representing lightness/brightness, redness/greenness, and yellowness/blueness, respectively) [17]. The corresponding values of a white standard were L* = 93.71, a* = −1.21, and b* = 0.48.

#### 2.6.2 Acid value and free fatty acids (FFAs)

The acid value and FFA of the oils were estimated according to the method described by Chaijan et al. [26] with slight modifications. One gram of oil was dissolved in ten volumes of hexane. To this, two drops of phenolphthalein indicator were added and titrated against 0.02 N potassium hydroxide (KOH) solution until the pink color was obtained. The acid value was expressed as mg KOH consumed to neutralize the free acids in 1 g of oil and calculated using the following formula:

(3) \[{\mathrm{Acid\; value}}=\frac{56.1\times N\left\times ({V}_{{\mathrm{s}}}\left-{V}_{{\mathrm{b}}})}{W},]\]

where *N* is the normality of KOH, *V*_s_ is the volume of KOH required for the sample, *V*_b_ is the volume of KOH required for blank, and *W* is the weight of the sample (in g).

FFA was expressed as % oleic acid present in the oil sample, calculated using the following formula:

(4) \[{\mathrm{FFA}}(\left \% )=\frac{{\mathrm{Acid\; value}}\times {M}_{{\mathrm{w}}}{\mathrm{of\; oleic\; acid}}\times 10}{{M}_{{\mathrm{w}}}{\mathrm{of\; KOH}}\times 1,000},]\]

where *M*_w_ is the molecular weight.

### 2.7 Oxidative stability of oil

#### 2.7.1 Peroxide value (PV)

The PV of oil was determined spectrophotometrically, as described by Bruno et al. [27], using 20 mM ferric thiocyanate as a reducing agent. A standard curve was plotted using cumene hydroperoxide at 0–50 ppm concentration, and the PV was expressed as meq O_2_/kg of oil sample.

#### 2.7.2 TBA reactive substances (TBARS)

TBARS analysis was performed as described by Bruno et al. [27] and Dave et al. [23], with slight modifications. Oil (10 mg) was thoroughly mixed with 2.5 mL of a TBA reagent (0.375% TBA, 15% TCA, and 0.25 M HCl). The mixture was incubated at 95℃ for 10 min. The reaction was stopped by cooling the tubes under running water and centrifuging at 3,600 × *g* for 20 min at 25℃. The upper layer was collected, and absorbance was measured at 532 nm. The TBARS content of the oil was measured using a standard curve plotted with 1,1,3,3-tetraethoxypropane and expressed as mg malonaldehyde (MDA)/kg oil.

### 2.8 Characterization of oil

To characterize the oil, four oil samples were selected as follows: 1. The sample was selected based on the highest yield with desirable oil quality (optimized condition), 2. Run 10 was selected as a negative control, which had lower oil quality (higher acid value, higher FFA, higher PV, and higher TBARS). The oil recovered via solvent extraction, i.e., 3. chloroform:methanol (2:1, v/v), and 4. hexane was taken as a positive control.

#### 2.8.1 Fatty acid composition

Fatty acid methyl esters (FAMEs) were prepared according to the method described by Muhammed et al. [28]. The derivatized FAMEs (1 μL) were injected into a gas chromatography (GC) (7890B series; Agilent Technologies, Santa Clara, CA, USA) equipped with a flame ionization detector and an HP 88 capillary column (J & W Scientific Column from Agilent Technologies). The GC conditions were set according to the manufacturer's instructions. Peak identification was based on the retention time of the Supelco^®^ external standard (37 Component FAME Mix). The identified peaks were integrated and calibrated against the standard curve using Open LAB CDS software (Chem Station edition; Agilent Technologies, Santa Clara, CA, USA) [23].

#### 2.8.2 Volatile compounds

Volatile compounds were measured using a GC-mass spectrometer (GC-MS) (HP 5890 series II; Hewlett Packard, Atlanta, GA, USA) equipped with an HP 5972 quadrupole mass detector, as outlined by Moula Ali et al. [29]. The spectra were obtained at an ionization energy of 70 eV. Ionization was performed at 250°C in electron ionization (EI) mode. The acquisition was monitored at a scanning rate of 0.220 s/scan at 25–500 amu. The ChemStation Library was used to identify volatile compounds (Library No. Wiley 275. L). The volatile compounds are expressed in abundance based on the peak area [23].

### 2.9 Statistical analysis

All the experiments were conducted in triplicates. BBD with three factors was used, and a second-order polynomial model was studied to calculate the *F*-ratio, coefficient correlation, and lack-of-fit test. The optimal conditions were extracted using the numerical optimization toolbox of Design-Expert 12.0.3. Duncan’s multiple range test enumerated the significant difference between mean values for fatty acid compositions and volatile compounds.

## 3 Results and discussion

The proximate composition of catfish species *C*. *magur* viscera contained 54.67% ± 0.13% moisture, 12.21% ± 0.57% proteins, 24.35% ± 0.76% fat, and 1.86% ± 0.51% ash (wet weight basis). Similar catfish species, i.e., *C*. *magur* from Kenya, were reported to contain 38.20% fat, which was relatively higher than those from Thailand [30]. The difference in total fat could be possibly due to the difference in geographical distribution and fish feed [31].

### 3.1 Extraction of oil

#### 3.1.1 Second-order polynomial distribution

Catfish visceral oil recovery optimization studies were carried out using BBD. After the experimental runs, the optimal conditions were developed using software, and the oil was extracted under optimal conditions and was used for further analysis (Table 2). The results for ANOVA are shown in Table 3. The *F*-values of each response revealed that the quadratic model was significant (*p <* 0.05). The *R* ^2^ value for each response under study was >0.95 (except for color a*, b*), suggesting that more than 95% of results matched the predicted values. According to Ferreira et al. [32], a model is adequate when *R* ^2^ > 0.95. This study’s lack of fit, which measures the model’s fitness, was insignificant (*p* > 0.05). Therefore, the number of experiments was sufficient to determine the effects of the independent variables on catfish viscera oil yield.

**Table 2:** Yield, recovery, color, and quality parameters of catfish viscera oils recovered using the wet rendering method

**Run**	**Oil yield** ^**a**^	**Oil recovery (%)**	***L*** *	***a*** *	***b*** *	**Δ** ***E*** *	**Acid value** ^**b**^	**FFA** ^**c**^	**PV** ^**d**^	**TBARS** ^**e**^
1	11.25 ± 0.29	47.06 ± 0.44	62.17 ± 0.10	−1.91 ± 0.02	19.18 ± 0.07	36.70 ± 0.19	5.23 ± 0.01	0.26 ± 0.04	0.532 ± 0.002	0.45 ± 0.04
2	11.28 ± 0.32	47.12 ± 0.31	62.15 ± 0.12	−1.90 ± 0.01	19.12 ± 0.04	36.67 ± 0.17	5.26 ± 0.01	0.26 ± 0.04	0.527 ± 0.001	0.45 ± 0.04
3	6.41 ± 0.74	28.13 ± 1.41	65.11 ± 0.13	−1.95 ± 0.03	19.21 ± 0.04	34.23 ± 0.22	5.25 ± 0.25	0.26 ± 0.01	0.566 ± 0.004	0.36 ± 0.04
4	5.64 ± 0.29	17.04 ± 0.33	68.19 ± 0.22	−1.93 ± 0.04	19.38 ± 0.01	31.80 ± 0.03	4.95 ± 0.01	0.25 ± 0.05	0.426 ± 0.004	0.40 ± 0.03
5	11.37 ± 0.78	45.42 ± 0.97	61.44 ± 0.21	−1.86 ± 0.02	19.11 ± 0.05	37.29 ± 0.42	5.24 ± 0.27	0.26 ± 0.01	0.433 ± 0.002	0.45 ± 0.02
6	7.10 ± 0.33	29.6 ± 0.53	63.21 ± 0.13	−1.93 ± 0.03	19.11 ± 0.04	35.78 ± 0.27	5.41 ± 0.28	0.27 ± 0.01	0.410 ± 0.001	0.41 ± 0.02
7	8.87 ± 0.21	36.13 ± 0.63	63.23 ± 0.12	−1.88 ± 0.04	19.21 ± 0.02	35.81 ± 0.15	5.42 ± 0.18	0.27 ± 0.05	0.396 ± 0.002	0.32 ± 0.01
8	11.25 ± 0.62	47.06 ± 0.44	62.16 ± 0.14	−1.90 ± 0.02	19.21 ± 0.07	36.74 ± 0.19	5.21 ± 0.01	0.26 ± 0.04	0.536 ± 0.002	0.45 ± 0.04
9	7.23 ± 0.41	29.69 ± 0.57	67.23 ± 0.12	−1.91 ± 0.03	19.41 ± 0.04	34.63 ± 0.15	5.11 ± 0.28	0.26 ± 0.01	0.418 ± 0.001	0.34 ± 0.02
10	10.93 ± 0.27	44.88 ± 0.48	61.21 ± 0.14	−1.84 ± 0.04	19.21 ± 0.02	36.55 ± 0.32	5.44 ± 0.01	0.27 ± 0.04	0.395 ± 0.003	0.32 ± 0.03
11	3.68 ± 0.29	13.92 ± 0.22	66.24 ± 0.41	−1.91 ± 0.03	19.51 ± 0.02	33.46 ± 0.24	4.83 ± 0.07	0.24 ± 0.03	0.492 ± 0.003	0.31 ± 0.04
12	7.32 ± 0.58	29.28 ± 0.51	65.24 ± 0.11	−1.90 ± 0.02	19.25 ± 0.01	34.32 ± 0.31	4.83 ± 0.27	0.24 ± 0.01	0.459 ± 0.002	0.30 ± 0.03
13	11.52 ± 0.79	47.31 ± 1.19	61.21 ± 0.32	−1.90 ± 0.01	19.17 ± 0.02	37.52 ± 0.10	5.25 ± 0.26	0.26 ± 0.01	0.373 ± 0.001	0.51 ± 0.03
14	6.69 ± 0.31	28.41 ± 0.97	67.12 ± 0.14	−1.93 ± 0.04	19.35 ± 0.02	32.65 ± 0.13	5.25 ± 0.26	0.26 ± 0.01	0.377 ± 0.002	0.46 ± 0.03
15	9.17 ± 0.64	21.19 ± 0.33	64.11 ± 0.13	−1.95 ± 0.03	19.21 ± 0.04	35.07 ± 0.15	5.34 ± 0.01	0.27 ± 0.04	0.470 ± 0.003	0.31 ± 0.03
Optimum conditions	12.34 ± 0.34^A^	50.67 ± 0.81^A^	61.48 ± 0.33^A^	−1.86 ± 0.03^A^	19.11 ± 0.02^C^	37.29 ± 0.25^B^	5.44 ± 0.21^B^	0.27 ± 0.02^B^	0.395 ± 0.001^A^	0.44 ± 0.02^B^
A	16.68 ± 0.51^B^	68.50 ± 0.73^B^	77.28 ± 0.04^B^	−1.88 ± 0.02^B^	17.86 ± 0.03^A^	24.12 ± 0.13^A^	2.34 ± 0.21^A^	0.12 ± 0.02^A^	0.366 ± 0.001^A^	0.05 ± 0.02^A^
B	13.54 ± 0.39^A^	51.49 ± 0.61^A^	78.11 ± 0.10^B^	−1.86 ± 0.03^A^	18.35 ± 0.01^B^	23.92 ± 0.05^A^	2.21 ± 0.18^A^	0.11 ± 0.01^A^	0.366 ± 0.002^A^	0.03 ± 0.01^A^

Solvent extraction methods were positive controls, i.e., hexane and chloroform:methanol.

^a^Oil yield in terms of g/100 g, based on wet weight; ^b^acid value in terms of mg KOH/g of oil; ^c^FFAs in terms of % oleic acid; ^d^PV in terms of meq O_2_/kg oil; ^e^TBARS in terms of mg MDAEq/kg of oil.

Runs 1-15, oil extracted using the wet rendering method.

Optimum condition: viscera to water ratio, 1:0.5 (w/v); temperature, 90℃; and time, 20 min.

*A*: Oil extracted with chloroform:methanol (2:1, v/v).

*B*: Oil extracted with hexane.

Mean ± SD from triplicate determinations.

Different uppercase superscripts in the same column indicate significant differences (*p* < 0.05) between *A*, *B,* and the optimum condition.

**Table 3:** ANOVA and regression coefficients of the second-order polynomial model for response variables against yield, recovery, color, and quality parameters of catfish visceral oils

Source	df	*p* value							*F* value						
		Oil yield	Oil recovery	L*	Acid value	FFA	PV	TBARS	Oil yield	Oil recovery	L*	Acid value	FFA	PV	TBARS
Model	9	<0.0001	<0.0001	0.0060	0.0002	0.0093	0.0001	0.0207	100.31	100.29	12.73	50.07	10.48	64.99	7.29
*A*	1	0.0005	0.0005	0.0148	0.0027	0.8394	0.0015	0.0838	64.00	64.00	13.30	30.31	0.0456	40.01	4.64
*B*	1	<0.0001	<0.0001	0.0006	<0.0001	0.0007	0.0123	0.3572	272.35	272.26	59.01	269.40	55.25	14.64	1.03
*C*	1	<0.0001	<0.0001	0.0180	0.0006	0.0021	0.0001	0.0838	216.39	216.33	11.98	60.70	34.15	107.15	4.64
*AB*	1	0.2307	0.2306	0.5464	0.6778	0.9223	0.0012	0.0030	1.86	1.86	0.4180	0.1942	0.0105	43.93	28.91
*AC*	1	0.1017	0.1019	0.0870	0.5823	0.9713	0.0002	0.1057	4.01	4.00	4.51	0.3453	0.0014	88.98	3.89
*BC*	1	0.5255	0.5233	0.0474	0.0023	0.1269	0.0036	0.0077	0.4652	0.4704	6.84	32.83	3.35	26.40	18.50
*A*²	1	0.0023	0.0023	0.1318	0.0205	0.4719	0.0014	0.3369	32.34	32.31	3.24	11.16	0.6049	40.51	1.13
*B*²	1	<0.0001	<<0.0001	0.0173	0.0058	0.5230	<0.0001	0.2720	178.63	178.58	12.25	21.26	0.4712	175.57	1.52
*C*²	1	<0.0001	<0.0001	0.0665	0.0060	0.5992	0.0003	0.2187	175.97	176.00	5.47	20.83	0.3143	82.25	1.98
Lack of Fit	3	0.9986	0.9985	0.2306	0.3094	0.0534	0.1500	0.1010	0.0086	0.0087	3.49	2.38	17.90	5.83	9.06
*R* ^2^	0.9945	0.9945	0.9582	0.9227	0.935	0.9902	0.9902	0.9915							
Adj. *R* ^2^	0.9846	0.9846	0.8829	0.7645	0.7520	0.9725	0.9725	0.9763							
CV %	3.89	3.89	1.10	1.53	1.46	1.47	1.47	5.48							

*A* (viscera: water, w/v), *B* (temperature), and *C* (time) represent independent variables.

*p*-values < 0.05 indicate that model terms are significant.

*F*-values of each response revealed that the quadratic model was significant (*p <* 0.05).

Lack of fit, which measures the model’s fitness, was insignificant (*p* > 0.05).

#### 3.1.2 Optimization of extraction

A numerical optimization toolbox was used to optimize the wet rendering conditions. According to the second-order polynomial equation, the optimum conditions for oil yield included viscera to water ratio, 1:0.5 (w/v); temperature, 90℃; and time, 20 min, with a desirability of 0.842. Under these conditions, the predicted oil yield was 12.34 g/100 g. The results revealed that the experimental value for oil yield was 12.40 g/100 g, demonstrating the high desirability.

#### 3.1.3 Effect of independent variables on the responses

##### 3.1.3.1 Extraction yield

The extraction yield of oil and recovery of oil ranged from 3.39 to 12.28 g/100 g and 13.92 to 50.43%, respectively. This change in values of both parameters suggested that all three independent variables were at linear and quadratic levels (Table 3). The independent variable, viscera to water ratio (w/v), demonstrated that with an increase in water ratio, a significant (*p <* 0.05) decrease in extraction yield and recovery was observed (Table 4). Similarly, Mbatia et al. [33] reported that the oil yield decreased with an increase in water content, which might be due to the formation of emulsion resulting in the entrapment of oil. In contrast, the effect of temperature showed an increase in the extraction yield at a temperature from 50 to 100℃. Similarly, El-Rahman et al. [22] observed an increase in oil yields up to 100℃ during wet reduction of tilapia and mackerel viscera. Further increase in temperature from 100 to 120℃ resulted in a significant (*p <* 0.05) decrease (73%) in the extraction yield (Tables 2 and 4). Further, increasing the temperature to 121℃ might have led to higher protein denaturation exposing the hydrophobic domain, resulting in protein–lipid interaction and decreased yield [8]. A study on the effects of varying heat treatments on lipid removal during pelagic fishmeal production observed that an increase in temperature above 85℃ could affect the lipid extraction efficiency [34]. Time factor exhibited a linear correlation with oil recovery. The increase in the extraction time increased the oil yield and oil recovery. Kudre et al. [35] also observed that a more extended extraction period effectively increased the yield. This could be correlated with the higher breakdown of adipose tissues with respect to time and the release of more fat, thereby improving the recovery [20]. It was noted that when compared to the chloroform:methanol (2:1, v/v) extraction method, the optimal conditions had a relatively lower oil yield and recovery (2 and 7%, respectively) (Table 2). The results indicated that the values were comparable and not very low with the prospect of any extraction method compared to the standard solvent extraction method.

**Table 4:** Effect of individual independent variables on the oil yield, oil recovery, color, and quality parameters of the catfish visceral oil using the wet rendering method

Process variables	Oil yield (g/100 g)	Oil recovery (%)	L*	Acid value (mg KOH/g of oil)	FFA (% oleic acid)	PV (mg cumene hydroperoxide equivalent/kg of oil)	TBARS (mg MDAEq/kg of oil)
*A*	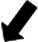	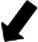	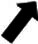	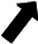	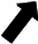	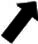	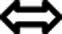
*B*	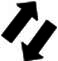	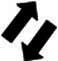	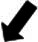	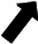	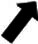	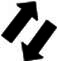	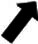
*C*	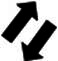	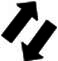	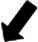	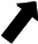	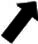	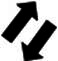	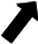

*A* (viscera: water, w/v), *B* (temperature), and *C* (time) represent independent variables.

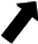
: increasing trend.

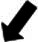
: decreasing trend.

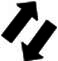
: increasing followed by decreasing trend.

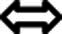
: Non-significant changes.

##### 3.1.3.2 Color

The color values of oils from the solvent extraction and wet rendering processes are shown in Table 2. During the wet rendering, the L* (lightness) values ranged from 61.21 to 67.23. These values were significantly (*p <* 0.05) influenced by all three independent variables at a linear level, as shown in Table 3. When more water was used during extraction, the resulting oil had a lower lightness value (L*). It is unclear why this happened, but this could be due to the difference in the oil’s chemical composition. On the other hand, the lightness value decreased as the extraction process continued with an increase in temperature and time. Many researchers have suggested various explanations for this phenomenon. Aidos et al. [36] reported that protein and carbohydrates in viscera hydrolyzed to smaller fractions and mixed in oil to lower lightness. Another study by Grebenteuch et al. [37] reported that high temperatures could polymerize browning substances by condensation and dehydration of the secondary products from lipid oxidation during the wet rendering method. However, no significant difference was observed in other color parameters, including a* values (−1.83 to −1.99) and b* values (19.11 to 19.51), suggesting no difference in greenness and yellowness of the oil, irrespective of the extraction methods used.

##### 3.1.3.3 Acid value and FFA

The acid value and FFA content are shown in Table 2. Among all the samples, the highest acid value of the wet rendering was from run 10 (5.44 mg KOH/g of oil), which was significantly higher than chloroform:methanol (56.98%) and hexane (59.37%) (*p <* 0.05). The optimal conditions exhibited lower acid values when compared to the chloroform:methanol and hexane method, i.e., 20.6 and 21.3%, respectively. The acid value is an index for the hydrolytic rancidity of fats and oils [38]. The increased acid value denotes a greater proportion of FFA, released due to hydrolysis from the glycerol backbone due to the extraction process, which is readily available for oxidation [39,40]. As determined, the FFA content was higher in the wet rendering from run 10, which was 20.8% higher than chloroform:methanol and 21.8% higher than hexane (*p <* 0.05). The acid value and FFA were significantly (*p <* 0.05) affected by all three independent variables at linear and quadratic levels (Table 3). It was observed that with an increase in water ratio, temperature, and time, there was a gradual increase in the acid value and FFA. This could be due to increased hydrolysis of fat and oil to release FFA [41,42]. Similar findings were observed by Suseno et al. [43] during the wet rendering of tilapia byproducts.

##### 3.1.3.4 PV

Generally, fish oil contains a high amount of PUFA, which can react with atmospheric oxygen, especially during extraction, and produce primary and secondary oxidation products. The presence of heavy metals in unrefined fish oil can intensify these oxidation reactions, as these metals often act as catalysts, accelerating the formation of hydroperoxides and other oxidative products [44]. It was observed that the PV of both the solvent-extracted oils remained lower when compared to all the runs in the randomized design and optimal conditions. It was observed that the PV of both the solvent-extracted oils remained lower when compared to all the runs in randomized design and optimal conditions. The PV of optimal conditions was found to be 1.07 times higher than oil recovered through solvent extraction (*p <* 0.05). However, all oil samples showed the PV within the limit described by the Codex Alimentarius Commission, i.e., <5 meq O_2_/kg of fish oil [45]. Furthermore, it was also observed that the PV had a linear relation with the water ratio during the experiment. The higher water content has been reported to hydrolysis triglycerides and liberate FFAs, which can easily undergo oxidation reactions and contribute to PV [43]. Moreover, the temperature also shows a profound effect on PV in the wet rendering process at 50–80℃. With the increase in temperature, the thermal degradation and oxidation of lipids are higher, which can react with oxygen more effectively [46,47]. Further, with an increase in temperature (80–121℃), the PV reduced, but an increase in TBARS was observed. This could be due to the reduction of primary oxidation products and an increase in the formation of secondary oxidation products [48,49]. Charuwat et al. [50] documented that the hydroperoxide decomposition rate may increase at a higher temperature, producing secondary compounds (aldehyde and ketone). Probably, extraction time exhibited a similar pattern for PV and TBARS, i.e., from 5 to 15 min, a slight increase in PV was observed, and from 15 to 25 min, PV decreased (Table 4). This can be supported by the hydrolysis of triglyceride ester bonds occurring more at prolonged extraction time, elevating the FFA level, which reacts with oxygen and generates hydroperoxides [36]. Weber et al. [51] also observed decreased PV during boiling silver catfish fillets for 30 min. This could be due to prolonged cooking time, where the initial hydroperoxides might decompose into volatile and non-volatile products.

##### 3.1.3.5 TBARS

The results for TBARS are presented in Tables 2 and 4. TBARS of oil extracted using the wet rendering method was higher, which was 8.8-fold higher than chloroform:methanol (2:1, v/v) and 14.66-fold higher than oil recovered through hexane (*p <* 0.05) under optimal conditions. These results correlated with PV, as discussed in the previous section. The high-value TBARS might be due to higher thermal degradation of fatty acids during wet rendering, resulting in more secondary oxidation products like MDA. These results were in line with that of volatile compounds detected in the oil samples. The results indicated that with an increase in temperature and time, a gradual increase in TBARS was observed (*p <* 0.05). In comparison, the water ratio did not affect the increasing trend of TBARS values. Weber et al. [51] also observed that thermal processing like boiling increases the TBARS of silver catfish fillets. Prolonged cooking may convert the primary oxidation products into secondary oxidation products, like aldehydes, i.e., MDA. Similar results were observed by Sajib et al. [52], where the TBARS increased during the prolonged cooking of herring fish fillets for 30 min to extract the fish oil.

### 3.2 Characterization

#### 3.2.1 Fatty acid composition

The fatty acid compositions of all four oil samples selected for characterization are given in Table 5. Linoleic acid was the predominant fatty acid in all four samples, accounting for about 25% of the total fatty acids. The second-highest fatty acid found in all oil samples was oleic acid, accounting for about 21% of total fatty acids. Sathivel et al. [20] also observed the predominance of omega-6 and omega-9 in catfish oils. The other fatty acids were found to be in moderate or lower concentrations. In the present study, it was also noted that the total amount of PUFA was higher (32–63%) than the total amount of saturated fatty acid (*p <* 0.05). Among PUFA, EPA and DHA were found at lower concentrations in all samples. Sathivel et al. [53] observed that the long-chain omega-3 fatty acids in catfish oil were significantly less (ranging from 1.8 to 2.1 mg/g). Based on the type of extraction methods, it was observed that oil recovered through hexane had the highest content of PUFA followed by chloroform:methanol (2:1, v/v), optimal conditions, and Run 10 (*p <* 0.05). The thermal process during wet rendering profoundly affected the reduction of the PUFA [54], but the optimal conditions retained the PUFA to a considerable quantity. Similarly, the extraction can also affect the recovery of PUFA; a longer extraction time can degrade PUFA to a more significant extent [55]. The oil obtained under optimal conditions showed 27.40 μg/g of oil EPA and 30.80 μg/g of oil DHA, which was significantly (*p <* 0.05) higher than the oil recovered by Run 10 (13.32 μg/g EPA and 21.12 μg/g of DHA, respectively). This can also be due to the higher rate of oxidation in oil recovered through Run 10, thereby degrading these compounds to produce the secondary oxidation compounds. These results were in line with the findings of Dave et al. [25].

**Table 5:** Changes in the fatty acid composition of catfish viscera oils recovered using the wet rendering method compared to two solvent extraction methods

Fatty acids	*A*	*B*	Optimal conditions*	Run 10**
	(μg/g of oil)			
C14	32.61 ± 0.02^a^	32.61 ± 0.01^a^	32.62 ± 0.01^a^	31.82 ± 0.02^b^
C14:1	3.32 ± 0.00^a^	3.32 ± 0.01^a^	3.51 ± 0.02^b^	ND
C15	4.61 ± 0.01^c^	4.51 ± 0.01^b^	4.61 ± 0.02^c^	4.22 ± 0.01^a^
C16	99.81 ± 0.21^a^	110.71 ± 0.32^b^	145.92 ± 0.29^d^	139.22 ± 0.30^c^
C16:1 (n7)	93.01 ± 0.31^c^	128.81 ± 0.23^d^	62.40 ± 0.24^b^	49.31 ± 0.21^a^
C17	4.12 ± 0.02^c^	4.22 ± 0.01^d^	4.01 ± 0.01^b^	3.71 ± 0.01^a^
C18	3.71 ± 0.02	3.72 ± 0.00^b^	3.61 ± 0.01^a^	3.61 ± 0.01^a^
C18:1 (n9)	249.80 ± 0.52^c^	256.81 ± 0.43^d^	229.90 ± 0.29^b^	201.22 ± 0.51^a^
C18:2 (n6)	305.11 ± 0.19^c^	324.32 ± 0.28^d^	262.90 ± 0.22^b^	212.51 ± 0.20^a^
C18:3 (n6)	0.72 ± 0.02^b^	0.81 ± 0.01^c^	0.61 ± 0.01^a^	ND
C18:3 (n3)	11.21 ± 0.01^c^	13.62 ± 0.02^d^	9.12 ± 0.02^b^	6.21 ± 0.02^a^
C18:4 (n3)	6.21 ± 0.01^c^	6.21 ± 0.02^c^	6.01 ± 0.01^b^	4.71 ± 0.03^a^
C20	1.91 ± 0.03^c^	2.02 ± 0.01^d^	1.71 ± 0.02^b^	0.51 ± 0.03^a^
C20:1 (n9)	12.92 ± 0.02^c^	13.71 ± 0.04^d^	10.11 ± 0.04^b^	7.41 ± 0.05^a^
C20:2 (n6)	3.71 ± 0.01^b^	3.71 ± 0.00^b^	3.71 ± 0.01^b^	2.32 ± 0.02^a^
C20:3 (n6)	2.41 ± 0.02^c^	2.42 ± 0.01^c^	2.31 ± 0.01^b^	0.60 ± 0.02^a^
C20:4 (n6)	7.52 ± 0.02^c^	7.82 ± 0.02^d^	7.31 ± 0.03^b^	5.80 ± 0.01^a^
C20:3 (n3)	0.22 ± 0.00^a^	0.22 ± 0.01^a^	0.20 ± 0.00^a^	ND
C20:4 (n3)	3.11 ± 0.03^b^	3.21 ± 0.02^c^	2.81 ± 0.02^a^	ND
C20:5 (n3)	29.11 ± 0.13^c^	29.41 ± 0.18^c^	27.40 ± 0.15^b^	13.32 ± 0.10^a^
C22	0.71 ± 0.00^a^	0.71 ± 0.01^a^	0.71 ± 0.01^a^	ND
C22:1 (n9)	2.12 ± 0.03^c^	2.22 ± 0.03^d^	1.92 ± 0.02^b^	0.51 ± 0.01^a^
C22:2 (n6)	6.82 ± 0.01^c^	7.11 ± 0.02^d^	6.02 ± 0.02^b^	4.20 ± 0.03^a^
C22:4 (n6)	1.21 ± 0.00^b^	1.31 ± 0.01^c^	1.22 ± 0.02^b^	0.41 ± 0.01^a^
C22:5 (n6)	2.41 ± 0.02^b^	2.41 ± 0.02^b^	2.51 ± 0.02^c^	1.31 ± 0.02^a^
C22:6 (n3)	33.21 ± 0.12^c^	34.91 ± 0.16^d^	30.80 ± 0.10^b^	21.12 ± 0.13^a^
C24:1 (n9)	0.91 ± 0.01^c^	0.82 ± 0.01^b^	0.71 ± 0.01^a^	ND
Saturated fatty acids	150.72 ± 0.20^c^	161.71 ± 0.02^d^	196.62 ± 0.16^b^	183.00 ± 0.01^a^
MUFAs	358.51 ± 0.31^c^	402.32 ± 0.22^d^	305.01 ± 0.02^b^	258.51 ± 0.20^a^
PUFAs	412.82 ± 0.32^c^	506.31 ± 0.28^d^	362.51 ± 0.17^b^	272.41 ± 0.11^a^
Total fatty acids	922.21 ± 0.10^a^	1070.31 ± 0.12^d^	864.41 ± 0.10^b^	713.92 ± 0.25^a^
Omega 6:Omega 3 (Ʃn6:Ʃn3)	3.92:1	3.75:1	4:1	5:1

*A*: oil extracted with chloroform:methanol (2:1, v/v), *B*: oil extracted with hexane.

*Oil extracted under optimal conditions: viscera: water (w/v) = 1:0.5, extraction temperature = 90℃, and extraction time = 20 min.

**Negative control, viscera: water (w/v) = 1:1.25, extraction temperature = 120℃, and extraction time = 25 min.

Values are given as mean ± SD (*n* = 3).

Different lowercase superscripts in the same row denote significant differences (*p* < 0.05).

ND: not detectable.

The omega-6 to omega-3 ratio is important in fish oil because these are two essential fatty acids the human body cannot produce on its own and must be obtained through diet. While omega-6 and omega-3 fatty acids are important for maintaining optimal health, a proper balance between them is crucial for overall health and well-being. According to the FDA, the optimal ratio of omega-6 to omega-3 fatty acids should be around 4:1 or lower [56]. A higher ratio of omega-6 to omega-3 fatty acids is associated with an increased risk of chronic diseases, such as heart disease, arthritis, and certain cancers [57]. In this study, the oil recovered through solvent extraction and optimal conditions showed an omega-6 to omega-3 ratio of 3.75:1 to 4:1. However, the oil recovered through Run 10 showed 5:1 omega-6 to omega-3 ratio, which made the oil undesirable for consumption.

#### 3.2.2 Volatile compounds

The volatile compounds in oils recovered through solvent and wet rendering methods are shown in Table 6. Aldehydes were found to be the most abundant volatile compounds in oils, including solvent extraction methods. Aldehydes have been widely used as indicators of lipid oxidation in various fats and oil-containing foods because they have low threshold values and mainly contribute to unpleasant odors [39]. The primary aldehydes identified in the present study were octanal, nonanal, pentanal, and hexanal, along with some minor aldehydes, i.e., 2,4-decadienal, and 17-octadecenal. The octanals are mainly produced due to omega-3 fatty acid oxidation, especially ALA [18]. The nonanal, pentanal, hexanal, and 2,4-decadienal are primarily derived from the oxidation of linoleic acid and oleic acid [58]. The oil extracted by solvent methods had fewer aldehydes than the wet rendering method. It can be noted that the temperature and time in the wet rendering process could affect the oxidation of oils. However, the oil obtained under optimal conditions had a low abundance of hexanal and 2,4-decadienal compared to Run 10.

**Table 6:** Changes in volatile compounds of catfish viscera oils recovered using the wet rendering method as compared to two solvent extraction methods

Volatile compounds	*A*	*B*	Optimal conditions*	Run 10**
**Aldehydes**				
Hexanal	ND	ND	0.24^b^	0.49^a^
Nonanal	ND	ND	0.01^b^	0.05^a^
2-Octenal	ND	ND	0.02^b^	0.06^a^
2,4-Heptadienal	ND	ND	0.03^b^	0.08^a^
Benzaldehyde	ND	ND	ND	0.07
2-Nonenal	ND	ND	0.03^b^	0.05^a^
2-Decenal	ND	ND	ND	0.10
10-Undecenal	ND	ND	ND	0.17
2-Dodecenal	ND	ND	ND	0.10
2,4-Decadienal	0.04	ND	0.96^b^	1.12^a^
Hexadecenal	ND	ND	ND	0.38
17-Octadecenal	ND	0.02	0.13^b^	0.19^a^
**Ketones**				
3,5-octadien-2-one	ND	ND	ND	0.02
**Hydrocarbons**				
Cyclohexene	ND	ND		0.02
Pentadecane	ND	ND	ND	0.06
Hexadecane	ND	ND	ND	0.02
Heptadecane	ND	ND	ND	0.08
**Others**				
Furan 2-pentyl	ND	ND	ND	0.02

*A*: oil extracted with chloroform:methanol (2:1, v/v), *B*: oil extracted with hexane.

*Oil extracted under optimal conditions: viscera: water (w/v) = 1:0.5, extraction temperature = 90℃, and extraction time = 20 min.

**Negative control, viscera: water (w/v) = 1:1.25, extraction temperature = 120℃, and extraction time = 25 min.

Values are expressed as abundance (×10^6^).

Different lowercase superscripts in the same row denote significant differences (*p* < 0.05).

ND: not detectable.

The highest number of volatile compounds found in oil recovered through Run 10 followed by optimal conditions. In the present study, the 3,5-octadien-2-one was found only in oil recovered by Run 10. 3,5-octadien-2-one is the most critical ketone produced from the lipid oxidation of omega 3 PUFA Field [59]. Similarly, the oil recovered from Run 10 showed the presence of furan derivatives, i.e., furan 2-pentyl (Table 6). Furan 2-pentyl can be generated from the 12-hydroperoxide of EPA and 16-hydroperoxide of DHA Field [60].

## 4 Conclusions

The oil recovery from catfish viscera was successfully carried out, which contained PUFA, including oleic acid, linoleic acid, ALA, EPA, and DHA. BBD studies were conducted using three independent variables, i.e., viscera to water ratio (w/v), extraction temperature, and time. The selected variables have shown a significant effect on the quality attributes such as color, acid value, FFA, PV, and TBARS, which was also confirmed by fatty acid analysis and oil volatile compounds. The optimal conditions (viscera to water ratio, 1:0.5 (w/v); temperature, 90℃; and time, 20 min) was developed with a comparable oil yield and quality to conventional solvent extraction methods. Finally, this study concludes that the acquired conditions are very well suited to extract the oil from *C*. *magur* viscera for application in the food industry aimed at a solvent-free extraction process. Further studies on the partial purification of this extracted oil with storage stability and application will be carried out.
